# Metabolomic Modularity Analysis (MMA) to Quantify Human Liver Perfusion Dynamics

**DOI:** 10.3390/metabo7040058

**Published:** 2017-11-13

**Authors:** Gautham Vivek Sridharan, Bote Gosse Bruinsma, Shyam Sundhar Bale, Anandh Swaminathan, Nima Saeidi, Martin L. Yarmush, Korkut Uygun

**Affiliations:** 1 Center for Engineering in Medicine, Harvard Medical School, Massachusetts General Hospital & Shriners Hospital for Children, 51 Blossom Street, Boston, MA 02114, USA; gvsridharan@gmail.com (G.V.S.); botebruinsma@gmail.com (B.G.B.); shyam.bale@gmail.com (S.S.B.); Nsaeidi@mgh.harvard.edu (N.S.); ireis@sbi.org (M.L.Y.); 2 Department of Control and Dynamic Systems, California Institute of Technology, Pasadena, CA 91125, USA; aswamina@caltech.edu

**Keywords:** metabolic networks, liver, cofactors, modularity

## Abstract

Large-scale -omics data are now ubiquitously utilized to capture and interpret global responses to perturbations in biological systems, such as the impact of disease states on cells, tissues, and whole organs. Metabolomics data, in particular, are difficult to interpret for providing physiological insight because predefined biochemical pathways used for analysis are inherently biased and fail to capture more complex network interactions that span multiple canonical pathways. In this study, we introduce a nov-el approach coined Metabolomic Modularity Analysis (MMA) as a graph-based algorithm to systematically identify metabolic modules of reactions enriched with metabolites flagged to be statistically significant. A defining feature of the algorithm is its ability to determine modularity that highlights interactions between reactions mediated by the production and consumption of cofactors and other hub metabolites. As a case study, we evaluated the metabolic dynamics of discarded human livers using time-course metabolomics data and MMA to identify modules that explain the observed physiological changes leading to liver recovery during subnormothermic machine perfusion (SNMP). MMA was performed on a large scale liver-specific human metabolic network that was weighted based on metabolomics data and identified cofactor-mediated modules that would not have been discovered by traditional metabolic pathway analyses.

## 1. Introduction

The application of large-scale -omics data has revolutionized our understanding of how complex cellular, tissue, and organ systems respond to various perturbations. Advancements in mass spectrometry have now enabled the simultaneous quantification of thousands of proteins [1,2] (proteomics) and small molecule metabolites [3,4] (metabolomics) in biological samples with great fidelity. These data provide a wealth of information to characterize physiological states and are increasingly used for drug discovery and basic science research [5] as well as for disease diagnostics [6]. However, the interpretation of these data to provide meaningful biological insight remains challenging due to their complex and multi-dimensional nature [7], thus necessitating the development of novel computational tools that integrate unbiased statistical analysis with annotated knowledge of biochemistry [8,9,10].

Metabolomics data, in particular, are now routinely utilized to study metabolism at the functional level, where biochemical substrates and products are quantified in samples obtained from in vitro cell culture [11], animal models [12], or patients in the clinic [13]. Relative concentration changes of metabolites in a biochemical reaction system between experimental conditions or time points can reveal how a system perturbation impacts well-known metabolic pathways as well as less obvious metabolic modules. However, identifying these pathway modules amidst a metabolic landscape comprising over 8000 reactions and 16,000 compounds (KEGG database [14]) has been challenging. While publicly available tools, such as Metaboanalyst (version 3.0) [10], provide an excellent rudimentary framework (KEGG) to identify known metabolic pathways enriched with statistically significant metabolites (SSMs), an investigator may seek to avoid the bias inherent within pathway enrichment analysis (PEA) and discover novel mechanisms driving a metabolic process. For example, metabolic modules that feature the shared production and consumption of energy cofactors (ATP, NADH, NADPH, etc.) provide a unique view of metabolic organization since they span multiple canonical biochemical pathways, and are particularly useful for the burgeoning field of organomatics [15]. In the context of liver transplantation, the reconstitution of energy charge potential (ratio of ATP relative to ADP and AMP) during ex vivo machine perfusion, for example, may serve as a benchmark criterion to evaluate whether or not the organ can sustain ischemia/reperfusion (I/R) injury and predict transplantation results [16]. As such, a thorough metabolomic characterization of human livers during machine perfusion [17] that highlights cofactor reconstitution could therefore potentially elucidate mechanistic insight into liver preservation and recovery. A better understanding of these mechanisms will facilitate the discovery of novel liver enzyme targets for customized therapy of livers prior to transplantation, such as de-fatting of steatotic ones, potentially using conjugated siRNAs that efficiently deliver to hepatocytes [18].

To facilitate the efficient discovery of these novel metabolic interactions, graph-based modeling of metabolic networks has emerged as a dominant computational platform for unbiased module detection and metabolomics data interpretation [19,20,21,22,23], and Frainay et al. have recently summarized many of these methods in a review [24]. Notably, Deo et al. recently presented a framework for identifying unbiased network modules enriched for changes in metabolite levels based on metabolomics data [25]. In addition, Jha and coworkers have also recently reported an elegant approach to identify sub-graphs in a bipartite metabolic network that maximized high-scoring nodes, where metabolite nodes with significant *p*-values (SSMs) received high scores [26]. Another way to abstract metabolic networks is as a reaction-centric graph where metabolites are treated as a shared resource, which provides an intuitive framework for facilitating the discovery of reaction modules that highlight the utilization of energy cofactors [27]. Another major challenge in executing module detection algorithms is the poor scaling of computational run time for large-scale networks. For example, the most comprehensive metabolic model, the Human Recon 2.1 [28], is a rigorously curated network comprising over 7000 reactions and node-pair calculations for this model can be computationally taxing for a single workstation. To alleviate the computational burden, cloud-based parallel computing [29] has recently emerged as a viable option to ensure that run-times for these graph-based algorithms on large-scale networks are within reason, thus obviating the need for cluster infrastructure within the lab or institution.

In this study, we developed a novel approach to metabolomics analysis coined Metabolomic Modularity Analysis (MMA), where we systematically partition a reaction-centric metabolic network into hierarchical modules enriched with SSMs using well established graph-based algorithms [30]. We applied MMA on the Human Recon 2.1 network model to analyze time-course metabolomics data collected from discarded human livers subject to three hours of ex vivo subnormothermic machine perfusion (SNMP) [17]. Machine perfusion is of clinical interest as a platform for the assessment and recovery of liver grafts prior to transplantation and time-course metabolomics analysis of perfused livers can elucidate metabolic modules implicated in the reversal of ischemia-induced injury. MMA on data collected from nine different livers identified predictable metabolic modules as well as less intuitive cofactor-based modules that are active during perfusion. In addition, comparison of modules across livers revealed functional differences, some of which were based on the warm ischemia time (WIT) or degree of steatosis. We demonstrate that MMA, owing to a graph edge-weighting scheme derived from SSMs based on metabolomics data, is advantageous over conventional PEA methodologies in its ability to elucidate network modules that span several canonical metabolic pathways.

## 2. Results

### 2.1. Human Recon 2.1 Network Topology Offers Insight into Metabolomics Data

**Figure 1:**
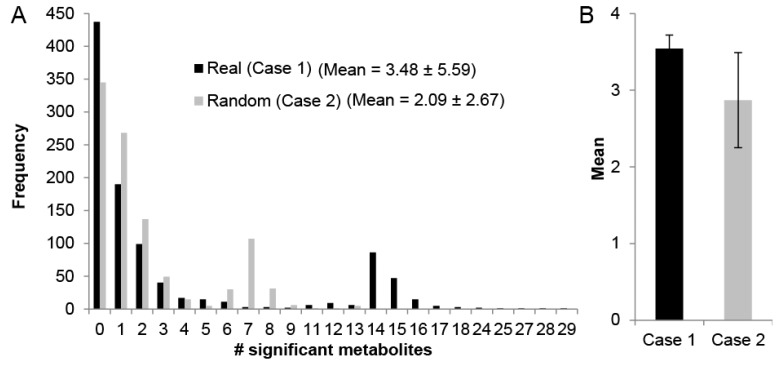
(**A**) significant metabolite distribution for *N* = 1000 randomly selected connected sub-graphs of 10 reaction nodes for the case where real metabolomics data for Liver 1 were used to determine significant metabolites by comparing metabolite levels between *t* = 0 to *t* = 3 h (Case 1) and where each of the 155 measured metabolites were randomly assigned significance with probability 73/155 (Case 2); (**B**) the distributions in (**A**) were obtained *N* = 25 times, and the mean of the distribution means were compared.

The use of MMA to discover novel modules that relate SSM hinges on the premise that the underlying metabolic phenomena driving the changes in metabolite levels can be captured by network topology, where SSMs are more likely to be connected on a metabolic network than are randomly selected metabolites. Therefore, we sought to first demonstrate that identifying sub-networks enriched with SSMs is actually biologically meaningful in the context of perfusion and not simply an effect of which metabolites were measured using mass spectrometry. To test if this is indeed the case, randomly connected sub-graphs were computed from the un-weighted Recon 2.1 reaction-centric network, and the number of SSMs in each connected sub-graph was computed for two cases. For case 1, the metabolites were assigned as SSMs based on Liver 1, where 73 of the measured 155 metabolites (see Methods) were found to be statistically significantly accumulated or depleted during the 3-h perfusion. For case 2, the SSMs were randomly generated where each of the 155 measured metabolites had an equal probability (*p* = 73/155) of being selected as an SSM. The distribution of the SSM frequency in these sub-graphs is compared for these two cases (Figure 1) with *N* = 1000 random sub-graphs of size 10 reaction nodes (manageable module size for metabolic analysis). The mean number of SSMs and standard deviation is 3.48 ± 5.59 for case 1 and 2.09 ± 2.67 for case 2 (Figure 1A), and both distributions are heavily skewed right and bimodal. The second peak in these bimodal distributions is due to sub-graphs containing reactions where several amino acids are reactants, such as protein synthesis or a generic human biomass reaction, which inflates the number of SSMs in the module. To statistically compare the means of these non-normal distributions, the sampling of *N* = 1000 sub-graphs was repeated 25 times to obtain normally distributed sample means. A comparison of these means finds the mean SSM frequency to be higher for case 1 (3.54 ± 0.18) than that of case 2 (2.87 ± 0.62) and that the difference is statistically significant (*p* < 10^−5^) based on a two-sample student’s *t*-test (Figure 1B). This suggests that the SSMs based on real metabolomics data collected from perfusion dynamics have a greater tendency to cluster within sub-graphs than do metabolites randomly selected to be SSMs, thus providing a basis for using systematic network analysis to aid in the discovery of biologically meaningful modules.

### 2.2. MMA Reveals Non-Intuitive Pathway Modules Engaged during Human Liver Perfusion

**Figure 2:**
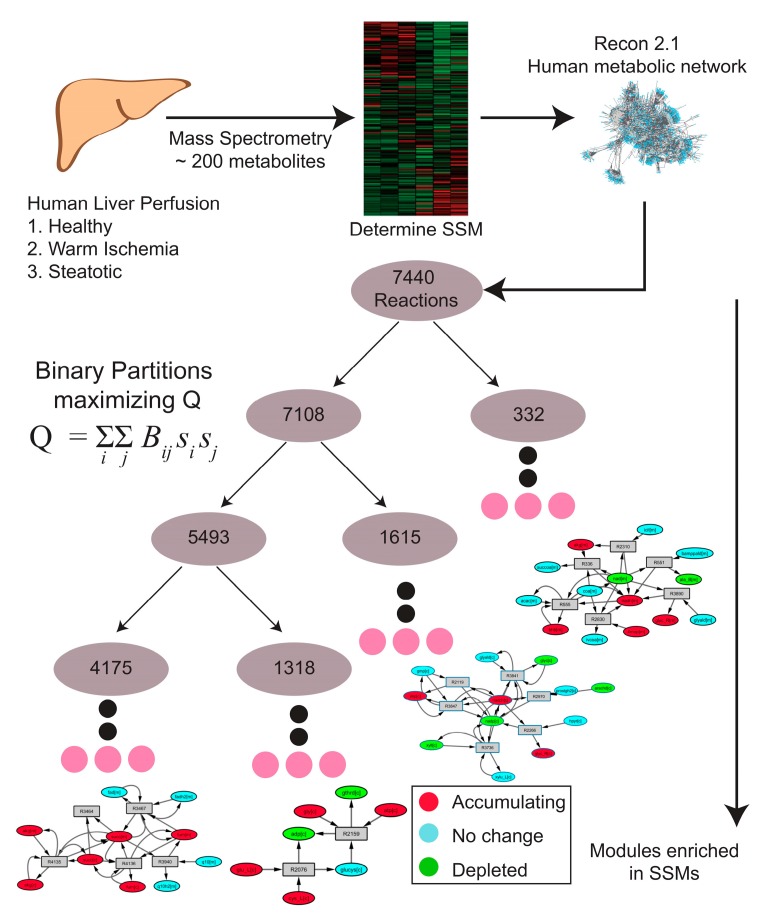
Schematic of the MMA workflow starting from sample collection to metabolomics analysis, to the partitioning of metabolomics-based weighted metabolic network using Newman’s algorithm, to discovering metabolic modules enriched with SSMs.

**Figure 3:**
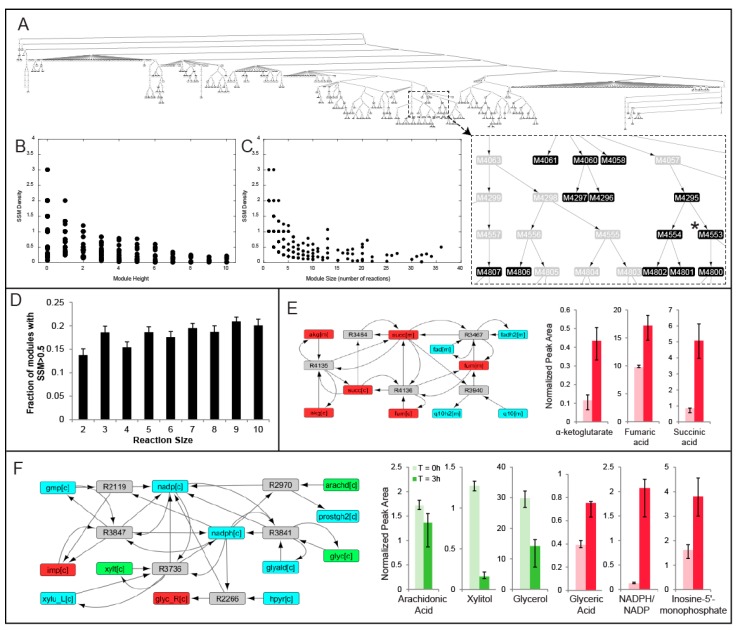
(**A**) MMA partition for Liver 1 where each node represents a module of reactions and the darker shaded nodes denote a higher statistically significant metabolite (SSM) density. For each module in Liver 1 with at least one significant metabolite, the SSM density is plotted against (**B**) hierarchical modular height and (**C**) module reaction size; (**D**) the fraction of modules with an SSM density greater than 0.5 is plotted against module reaction size. The modular compositions as well as the significantly elevated/depleted metabolites within the modules are both shown for an intuitive module that could have been predicted using PEA (**E**) and one where PEA would not have been effective at capturing the NADPH-mediated interaction (**F**). These modules were both derived from the partition of Recon 2.1 based on the metabolomics data collected from Liver 1. Red, green and blue nodes represent accumulating, depleting and non-significant/non-measured metabolites respectively.

MMA was performed on weighted reaction-centric graph networks derived from the Human Recon 2.1 model for each of nine livers, where metabolites were denoted as SSMs if there was a statistically significant change in the metabolite level between the *t* = 0 h and *t* = 3 h time points. The overall workflow of how MMA partitions a metabolic network into modules enriched in SSMs is outlined in Figure 2. For Liver 1, a ‘control’ liver (see Table 1), MMA partitioned the network into 5206 hierarchical modules, each module containing a subset of the original 7440 reactions. Furthermore, 577 of these modules possessed at least one reaction that involved the production or consumption of an SSM (Table 2). The hierarchical arrangement of these modules is shown in Figure 3A, where each node in the hierarchy represents a module of reactions. The SSM density, defined as the number of SSMs in a module divided by the number of reactions in the module, increases with greater depth in the hierarchy, which is measured by the ‘height’ of a module, or the farthest distance between that module and a terminal module (Figure 3B). As expected, the size of the modules also decreases with depth, so the smaller modules tend to have greater SSM density (Figure 3C). Similar trends were observed for the partitioning of all other liver-specific networks (unreported result).

In this study, we sought to focus on only a fraction of modules that are likely to be biologically significant based on the density of SSMs. We chose to examine modules comprising at least two reaction nodes with an SSM density greater than 0.5 (henceforth referred to as the baseline module criteria), ensuring at least one SSM for each reaction in the module on average. As a means to determine where an SSM density of 0.5 lies on the distribution of SSM frequency, *N* = 1000 random sub-graphs of size *N* (2–10) were computed (Methods), and the fraction of sub-graphs that satisfied the baseline module criteria was computed. That fraction was determined to have a range between 0.13 (*N* = 2) and 0.21 (*N* = 9) (Figure 3D) and the error bars represent standard deviations obtained from repeating the process ten times for each module size *N*. Using these SSM frequency distributions, one can assign a probability value for each module determined by MMA as the percent chance that a random connected sub-graph of the same size would have at least as many SSMs as the specified module.

For each module satisfying the baseline module criteria, pathway enrichment analysis (PEA) was performed (see Methods), where the number of modular unique SSMs belonging to each predefined KEGG pathway was computed (see Methods). In many cases, the number of SSMs in a specified module is equal to the maximum number of SSMs that belong to the same KEGG pathway. For example, Module 4283 for Liver 1 (node not shown in Figure 3A) involves alpha-keto glutarate, succinate, and fumarate, all of which are significantly accumulating during perfusion. The maximum number of these metabolites that belonged to the same KEGG pathway is 3, as they all participate in the pathway ‘TCA cycle’ (Figure 3E). In this regard, MMA is capable of identifying high SSM density modules that correspond to well-studied biochemical pathways, in addition to ones that span multiple canonical pathways and are less intuitive.

In other cases, the number of modular SSMs far exceeds the maximum number of SSMs that belong to the same KEGG pathway. For example, another module in Liver 1 (Module 4553, node shown in Figure 3A) is comprised of five reactions and captured the depletion of arachidonic acid, xylitol, and glycerol, as well as the accumulation of inosine-5-monophosphate, glyceric acid, and NADPH relative to NADP^+^ (Figure 3F). These reactions are catalyzed by xylulose reductase, hydroxypyruvate reductase, prostaglandin G/H synthase, glycerol oxidoreductase, GMP reductase, and guanosine-5-phosphate oxidoreductase and all involve the shared production and consumption of NADPH. However, the maximum number of SSMs from this module that belong to the same KEGG pathway is only 2, where glycerol and xylitol are both classified under ‘pentose and glucoronate interconversions’, suggesting that certain modules of reactions relating SSMs may not be intuitive by solely observing conventional biochemical pathways. Across all livers, an average of 40.41 ± 13.03 (mean ± standard deviation) percent of the modules satisfying the baseline module criteria involve these counter-intuitive interactions where the maximum number of SSMs belonging to the same KEGG pathway less than the number of SSMs in the module was computed (Table 3). The composition of all modules for each liver is available in Supplementary Data.

### 2.3. Comparison of Conserved Modules across Livers

**Figure 4:**
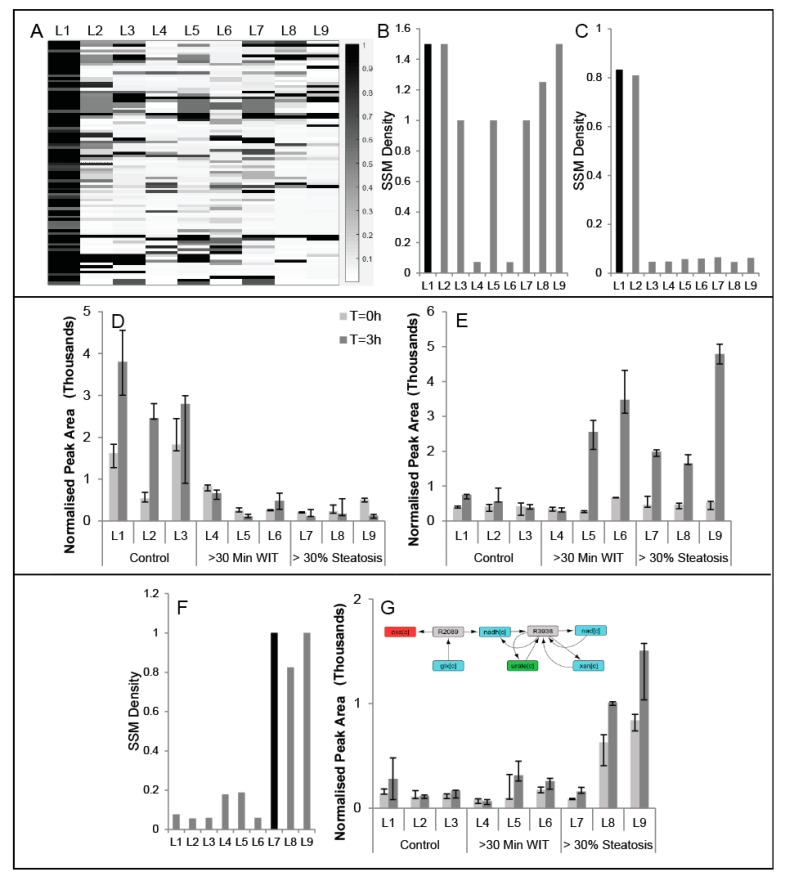
(**A**) heatmap where each row represents a Liver 1 module for which the SSM density is greater than 0.5 and each column represents each of the nine livers. The color entry in the heatmap, based on the colormap, denotes the largest SSM density for the module present in the partition of the column’s liver that contains all the reactions for the Liver 1 module in that row. The specific values of the SSM densities from the heatmap (**A**) are plotted for against liver number for modules (**B**) Liver 1, Module 3484 and (**C**) Liver 1, Module 4553. The lack of high SSM densities in all livers other than Livers 1 and 2 can be explained by the lack of significant metabolite changes in (**D**) glyceric acid and (**E**) inosine 5-monophosphate. (**F**) Liver 7, Module 884, an example of a module with an SSM density greater than 0.5 in only steatotic livers, largely explained by the accumulation of (**G**) oxalic acid.

A module observed as having a high SSM density for one liver may not be conserved in other livers, since metabolic dynamics during perfusion may vary depending on donor characteristics (Table 1). To quantify the extent to which a module is conserved, we compute the highest SSM density from a module in liver Y that contains all the reactions contained in the module from liver X. If the SSM density for the module in liver Y is also high (>0.5), then the module is more likely conserved; however, if the SSM density in Y is far lower, the module in Y is likely closer to the parent module in the hierarchy with many other reactions and a lower SSM density, suggesting that the module in X is not that well conserved in Y. For each module in liver 1, the highest SSM density of a module in each liver with the reactions contained in the liver 1 module is plotted as a heatmap (Figure 4A), revealing conservation of some modules across all the livers. For example, the reactions for glutathione synthesis, present in Liver 1—Module 3484, belong to modules with an SSM density of at least 1.0 in seven of the nine livers (Figure 4B). On the other hand, Liver 1—Module 4553 is conserved in Liver 2, but not in Liver 3, which is also a ‘control’ liver (Figure 4C). Examining the SSMs responsible for the module detection reveals that the changes in glyceric acid (Figure 4D) and inosine-5′monophosphate (Figure 4E) during are not statistically significant for Liver 3, possibly explaining why Liver 1—Module 434 does not have a corresponding module in Liver 3 or the other WIT/steatotic livers. In another example, a module found in a steatotic liver (Liver 7, Module 884) that contains glyoxylate oxidase and xanthine dehydrogenase is only identified in steatotic livers (Figure 4F) and can be explained by the fact that oxalate produced from glyoxylate is selectively accumulating during perfusion only in steatotic livers (Figure 4G).

### 2.4. Impact of Edge-Weights on Identifying Cofactor Modules

In the Methods section, we motivate the need to weight edges based on an example where a module enriched with SSMs would not be identified if all edges were treated with equal distance due to branching in the network. To further demonstrate the utility of weighting the graph edges based on Reaction Scores (Methods), MMA was also performed on the Recon 2.1 reaction-centric network where the edge between each node-pair was treated as equal and assigned the distance of 1.0. For each Liver 1 module satisfying the baseline modular criteria, the largest SSM density of the modules in the un-weighted network partition that contained all the reactions in the specified Liver 1 module was computed. Out of the 88 Liver 1 modules, only 13 had a corresponding module in the un-weighted case that satisfied the baseline modular criteria and none had an SSM density greater than that of the Liver 1 module (Table 4). These results suggest that more than 80% of the modules uncovered using MMA and weighted edges would not have been identified if all the edges were treated equally.

Furthermore, we tested the impact of edge-weighting on the ability of MMA to detect modules based on the shared production and consumption of four metabolic cofactors (NADPH, NADH, ATP, FADH_2_). For each liver’s partition, the number of modules satisfying the baseline modular criteria that involved the shared production/consumption of each cofactor was computed (Table 5). Cofactors from different compartments were all treated the same in enumerating the number of modules involved for each one. For modules that satisfied the baseline modular criteria in the partition of the un-weighted network, no modules were identified that capture the utilization of NADPH or FADH_2_ and only three and six modules involve ATP and NADH, respectively. In contrast, MMA-based partitioning with edge-weighting show an overall increase in the average number of cofactor-centric baseline criteria modules across all nine livers for NADPH (17.0 ± 6.08), FADH_2_ (2.0 ± 2.1), ATP (44.3 ± 18.4 ) and NADH (21.4 ± 13.0).

## 3. Discussion

The development of novel experimental and computational tools to understand metabolic changes at an organ level is important, particularly in case of transplantation. In this work, we introduce Metabolomic Modularity Analysis (MMA) as a novel approach to identify groups of reactions, or modules that elucidate the relationship between statistically significant metabolites (SSM) as a measure to evaluate the outcomes of machine perfusion on human livers. To our knowledge, we are the first to integrate metabolomics data with a reaction-centric graph representation of Human Recon 2.1, which is currently the most comprehensive annotated metabolic network describing human metabolism. Until recently, metabolomics data analysis has broadly fallen into two broad categories. The first involves the use of strictly data-driven machine learning techniques, where multivariate approaches such as principal component analysis (PCA) or linear discriminant analysis (LDA) can determine a set of metabolites that most contribute towards the separation of data between experimental groups in a multi-dimensional space. These approaches are un-biased and provide the investigator with a mathematical model relating metabolite levels and experimental outcome. In contrast, more ‘knowledge-driven’ metabolomics data analysis approaches involve determining which annotated biochemical pathways are affected by the experimental condition based on SSMs. However, pathway enrichment analysis (PEA) is inherently biased based on how each metabolite was previously classified into a traditional biochemistry pathway. In this regard, MMA offers the un-biased nature of data-driven methods while incorporating known biochemical interactions that are embedded in the graph network model.

While the integration of metabolomics with metabolic networks has been previously explored, a defining feature of MMA is the use of reaction-centric networks, where metabolites are treated as a shared resource. As a result, high-degree metabolites such as metabolic cofactors need not be restrictively assigned to one pathway as would be necessary with a bipartite graph representation with both reaction and metabolite nodes, provided each cofactor is only assigned one unique node. Reaction-centric graphs provide a flux-centric view of metabolism to capture interactions between reactions that are mediated by the production and consumption of cofactors, which we believe to be paramount to the study of metabolic systems. Another advantage of MMA is that its output is a hierarchical tree of modules, providing an investigator with a multi-resolution view of how the metabolic system is organized. For example, to determine how two modules of interest are related to each other, one can examine the common ancestor module in the hierarchy to discover mechanisms of feedback and cross-talk between the individual modular functions. Unfortunately, allosteric regulatory information is not included in the Recon 2.1 network model and therefore this information could not be incorporated into the construction of the weighted reaction-centric graph network. Going forward, databases such as BRENDA can be utilized to augment stoichiometric connections with known inhibitory and activating regulatory mechanisms [31].

One major hurdle for developing and using graph-based analysis on large metabolic networks has been the computational burden posed by graph algorithms that scale poorly. For example, the run-time for exhaustive node-pair calculations scale *O*(*n*^2^), limiting the usability of these algorithms to smaller size networks if only single machines are available. Parallel computing alleviates run-time burden by distributing repetitive computations onto multiple processors simultaneously. However, the average user may not have access to existing computer cluster infrastructure or user-friendly software for managing parallel operations on clusters. To address this, Mathworks^®^ (Natick, MA, USA) has recently introduced the integration of Matlab(c) Distributed Computing Server with Amazon’s Elastic Cloud Compute (EC2) service, allowing easy access to workers on the cloud and seamless conversion of for-loops to parallel for-loops (parfor loops). MMA is scripted to maximize parallel operations enabling the successful partition of the Recon 2.1 model in less than three hours using one cluster node (c3.8 × large) with 16 workers on Amazon EC2 (Table 2). In this study, we therefore demonstrate that-omics analysis using large-scale graph networks is operationally limited only by the cost of Matlab(c) server licenses and the rental of workers on Amazon (Seattle, WA, USA), which at current rates total to only $2.80/h for one cluster. The opportunity to seamlessly execute graph algorithms on large-scale metabolic networks offers a path towards the development of more complex multi-omic integration.

As a primary test case, we applied MMA on time-course metabolomics data collected from biopsy extracts on nine discarded human livers to determine which metabolic pathways are activated during a subnormothermic machine perfusion experiment (all perfusion metabolomics data and MMA-based modules for each liver are available in Supplementary Materials). MMA correctly identified known pathway modules that have been previously implicated in alleviating ischemic injury, such as the activation of the TCA cycle for reconstitution of ATP, glutathione synthesis to replenish depleted pools of the antioxidant. In addition, our finding that glycerol metabolism might be influenced by perfusion based on NADPH pools is corroborated by the fact that glycerol has been previously observed to be elevated in ischemic human livers and re-normalized post-perfusion [32,33]. More importantly, MMA discovered modules that would not have been identified using PEA based on pre-defined metabolic pathways catalogued in the KEGG database. We found that, across all livers, an average of 40.4% of the modules that satisfy the baseline modular criteria (at least two reactions and an SSM density greater than 0.5) did not have a corresponding KEGG pathway that contained all the metabolites in that module. Even if such a KEGG pathway existed, it may not highlight the same interaction between metabolites that was captured by the MMA module. For example, in Liver 1, Module 5024 contains two SSMs relating alpha-ketoglutarate and 3-hydroxybutyrate. The KEGG pathway ‘butanoate metabolism’ also contains these two metabolites, but relates them through eight reaction steps rather than the two reaction steps captured by MMA mediated by the production and consumption of mitochondrial NADH (Supplementary Figure S1).

An important aspect of MMA is that, in constructing the reaction-centric graph network, the edges are weighted such that node pairs with a relatively large SSM density are given a shorter edge to ensure the distance-based partitioning assigns them to the same module. We demonstrate that MMA-partitioning of weighted networks is beneficial for identifying cofactor mediated modules compared to the partitioning of un-weighted networks. Moreover, weighting edges facilitates the analysis of state-specific networks, where, in this case, each liver had a tailored reaction-centric network based on SSMs characterizing its perfusion dynamics. In this regard, we found some MMA modules unique to some livers but not conserved across all nine, which is driven by certain metabolites whose significant accumulation or depletion is selective to certain livers, sometimes based on donor characteristics such as the degree of steatosis. This state-specific analysis of liver metabolic pathways during perfusion can provide an investigator with additional insight as to how ischemic or steatotic livers differentially respond to machine perfusion; however, the current study is under-powered to report statistical differences between groups due to the general shortage of donor human livers for experimentation.

For simplicity, we have thus far treated all SSMs equally, as long as the difference in metabolite levels between the *t* = 0 and *t* = 3 h time points was statistically significant. A natural extension of this formalism would be to incorporate the magnitude of metabolite change into the Reaction Score computation, where metabolites with a larger fold-change would have a correspondingly increased contribution towards the value of the distance between the node pair. Moreover, the application of MMA extends beyond characterizing SSMs based on time-course metabolomics data. The algorithm can be applied to other metabolomics experimental designs where metabolites can be flagged as being an SSM. For example, if metabolomics data are collected from two experimental treatment groups obtained from in vitro cell culture or an in vivo animal model, differentially elevated or depleted metabolites can be treated as SSMs and MMA would reveal modules that characterize the metabolic effects of the experimental condition.

In conclusion, we demonstrate MMA to be a powerful approach for metabolomics analysis to identify counter-intuitive reaction modules that relate SSMs. The utility of MMA is to identify regions of a metabolic network that are perturbed as a result of an experimental condition. However, an investigator must resist the temptation to comment on the up/downregulation of reaction fluxes within the perturbed module based on the direction of metabolite levels. Intracellular metabolite concentration changes are insufficient to ascribe changes in reaction flux because an accumulating metabolite, for example, may be due to either an increased production flux or a reduced consumption flux. These metabolic fluxes can be more rigorously measured C^13^ isotopomer metabolic flux analysis [34], but the coverage of pathways is limited based on tracer design, availability, and cost. Flux balance analysis, an optimization technique used to analyze metabolic systems at steady state, could provide a less resource-intensive approach to arrive at an approximate flux distribution; however, many fluxes in a large scale network may not be resolved due to insufficient constraints [35]. Future work in the realm of organomatics research will entail the development of computational platforms for multi-omic data analysis, including transcriptomic, proteomic, metabolomics and even fluxomic data to better elucidate metabolic mechanisms involved in organ preservation and recovery.

## 4. Methods

### 4.1. Human Liver Perfusion

Human livers that were rejected for transplantation and consented for research were obtained from transplant centers affiliated with the New England Organ Bank, following the standard procurement protocol for orthotopic transplantation. Nine such livers were subject to subnormothermic machine perfusion (SNMP) as previously described by Bruinsma et al. [36]. Briefly, the livers out of ice were first flushed with Lactated Ringer’s solution and then perfused with a constant flow of nutrient rich and oxygenated media at room temperature through the portal vein and hepatic artery for three hours. Wedge biopsies were taken right before the perfusion and once every hour during perfusion and were used for metabolomics analysis. Of the nine livers chosen for SNMP, three experienced a warm ischemia time (WIT) of greater than 30 min, three exhibited greater than 30% macrovesicular steatosis, and the remaining three were considered control ‘healthy’ livers that should have been transplanted but were discarded for logistical reasons. The objective was to see how the state of the liver affected its dynamic metabolomics profile and if the data could provide novel insight into which metabolic modules are activated during perfusion.

This study (No. 2011P001496) was approved by the Massachusetts General Hospital Institutional Review Board (IRB) and the New England Organ Bank (NEOB), and we conducted these studies in accordance with IRB and NEOB approved guidelines. All donors provided informed consent for research on livers post-death, if unsuitable for transplantation.

### 4.2. Metabolomics Analysis 

Tissue biopsies were homogenized using a mortal/pestle in liquid nitrogen. Metabolomics analysis was performed on crushed tissue using two different analytical platforms. A 3200 QTRAP LC/MS-MS (AB Sciex, Foster City, CA, USA) system was used to quantify metabolic cofactors (ATP, ADP, AMP, NADPH, NADP, NADH, NAD, GSH, GSSG, FAD) in tissue metabolite extracts using the Multiple Reaction Monitoring (MRM) mode. Metabolites were extracted from approximately 25 mg of crushed tissue by adding 250 μL of a 2/1 (*v*/*v*) mixture of methanol/chloroform followed by three freeze–thaw cycles, where the tissue and solvent were rapidly frozen in liquid nitrogen for 30 s and thawed at room temperature. After each freeze–thaw cycle, the sample was vortexed for 10 s. After the final freeze–thaw cycle, 200 μL of ice cold water was added to each extract, followed by a 1 min centrifugation at 15,000 *g*. The extract forms a biphasic mixture, and the upper phase was transferred to an HPLC vial for LC/MS analysis. Redox ratios (e.g., NADPH/NADP^+^) were calculated based on the relative concentrations of each metabolite in the extract, which is determined by the peak area of each MRM transition and the corresponding calibration curve based on serial dilution of pure chemical standards. In addition to cofactor analysis, primary metabolites were quantified using GC-TOF-MS at the West Coast Metabolomics Center (Davis, CA, USA). Briefly, the homogenized samples were subject to metabolite extraction using a mixture of acetonitrile, isopropanol, and water followed by the spiking of internal standards to the extract sample. In total, 155 metabolites and 10 cofactors were quantified for *t* = 0 h and *t* = 3 h time-point biopsies for three technical replicates, where the extraction and MS analysis was performed individually for each replicate.

### 4.3. Statistically Significant Metabolites (SSM)

For each liver, metabolites were found to be statistically significantly accumulated or depleted by comparing the normalized peak areas between the *t* = 0 and *t* = 3 h time points (*p* < 0.05 Mann–Whitney-U test). For Liver 1, the liver with the least WIT, 73/155 of the metabolites measured by GC/MS were SSMs. The Recon 2.1 model incorporates compartmentalization and metabolites are denoted as belonging to either the extracellular space, cytosol, mitochondria, nucleus endoplasmic reticulum, peroxisome, lysosome, or the Golgi apparatus [28]. Since the metabolite extraction procedure is not specific to cytosolic extraction, we assumed that SSMs could be a result of any compartmental fraction. Therefore, if a metabolite was found to be an SSM, all compartmental versions of that metabolite in the Recon 2.1 model were treated as an SSM. As a result, for Liver 1, while only 73 unique metabolites were quantified as SSM, 129 of the 5063 metabolites in the model were denoted as SSM.

### 4.4. Bipartite Graph Construction

A bipartite graph, comprised of of both reaction and metabolite nodes, was constructed as a square directed connectivity matrix of size *N* + *M* where *N* is the number of reactions in the model and *M* is the number of metabolites. A directed edge was assigned from a reaction node to a metabolite node if the metabolite was a product of that reaction based on stoichiometry. Similarly, a directed edge was assigned from a metabolite node to a reaction node if the metabolite was a reactant for the reaction. If the reaction was listed as ‘reversible’ in the model, then both directions were considered in the assignment of edges. Metabolites whose production and consumption did not constitute a meaningful interaction between components were excluded from the bipartite graph. For example, if a hydrogen atom (H^+^) was produced by reaction *R_i_* and consumed by *R_j_*, the interaction between these reactions was based on an abundant inorganic ion was not considered significant. A full list of which metabolites in Recon 2.1 were excluded as not having a biologically meaningful interaction is included in Supplementary Materials. Along these lines, many investigators conducting metabolic network analysis have often excluded metabolic cofactors as well, such as ATP, NADH, NADPH, etc. However, our previous studies on metabolic modularity analysis suggested that interactions based on the production and consumption of cofactors revealed potentially significant interactions between reactions that spanned several canonical metabolic pathways. Therefore, cofactors involved in making reactions thermodynamically favorable were included in the bipartite network. The bipartite graphs, and sub-networks of the bipartite graphs were used for visualization of MMA-identified modules using Cytoscape software (version 3.0) [37].

### 4.5. Reaction-Centric Adjacency Matrix Computation

MMA performs a systematic partitioning of reactions in a reaction-centric network, where metabolites are treated as a shared resource. Reaction-centric graph networks were constructed using an un-directed square and symmetric adjacency matrix (RG) of size *N*. In the ‘unweighted’ case, an undirected edge between a reaction pair [*Ri*,*Rj*] was assigned as *RG*(*i*,*j*) = 1 if a metabolite produced by *Ri* was consumed by *Rj*. Computationally, this was performed using the bipartite graph network, from which a vector of all outgoing metabolites from *Ri* and incoming metabolites to *Rj* were computed, and if at least one metabolite was common between the two vectors an edge between the two reaction nodes was assigned. For MMA, the edges between two reaction nodes are weighted based on the density of SSMs participating in those reactions. This was done by first assigning a reaction score (*RS*) to each reaction node in the network as the number of SSMs that are either produced or consumed by that reaction. The edge distance for a reaction pair *RG*(*i*,*j*) was then assigned as the reciprocal of the sum of the reaction scores, if at least one of the reaction nodes had an *RS* > 0:

(1) $$RG\left(i,j\right)=\frac{1}{RS_{i}+RS_{j}}.$$

For the Human Recon model, this requires _7440_C_2_, or ~28 million computations, so parallel computing on Amazon EC2 was used to ease the burden of computational run time.

**Figure 5:**
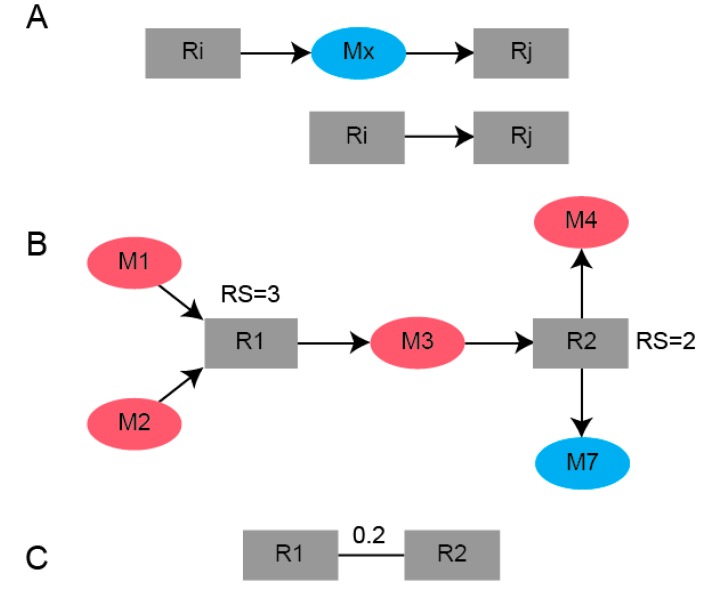
Schematic of the conversion of a (**A**) bipartite graph using (**B**) reaction scores computed from the number of SSMs either produced or consumed for a given reaction to a (**C**) weighted reaction-centric graph.

This is highlighted using the toy network example in Figure 5, where the distance between [*R_1_*,*R_2_*] is 0.2 because three SSMs are involved with *R_1_*, two SSMs are involved with *R_2_* and the distance is the reciprocal of that sum. Shorter distances between reaction nodes enable the clustering of reaction nodes that have a high SSM density. The importance of weighting edges to ensure the algorithm detects modules enriched in SSMs is highlighted by an example in Supplementary Figure S2.

It is important to note that, in metabolism, many bio-transformations are catalyzed by different enzymes and several enzymes are promiscuous and catalyze many different reactions. Recon 2.1 accounts for the former by denoting multiple reaction nodes for the same metabolite conversion of substrate to product, each catalyzed by a different enzyme. Regarding enzyme promiscuity, there are multiple cases in the model where different reactions have the same enzyme commission number listed. In this regard, our analysis is focused on the clustering of reaction nodes, rather than the enzymes themselves.

### 4.6. B-Matrix Construction

From the reaction-centric matrix (*RG*), a distance matrix (*D*) is computed where *D*(*i*,*j*) is the shortest path distance between *Ri* and *Rj*. Using the distance matrix, another matrix, B, is constructed such that reaction pairs [*Ri*,*Rj*] that have a relatively large distance *D*(*i*,*j*) will receive a negative *B*(*i*,*j*) and those with a short distance will receive a positive *B*(*i*,*j*). More specifically, *B*(*i*,*j*) is a value from between −1 and 1, computed from a linear regression based on the rank the node-pair distance falls amongst on a list of all node-pair distances the two nodes are involved in. The matrix *B* is used to determine modularity using Newman’s partition algorithm where reactions from reaction pairs with a large and positive *B*(*i*,*j*) are placed in the same module and those with large negative *B*(*i*,*j*) values are binned in separate modules. For each reaction pair [*Ri*,*Rj*], all distances *D*(*i*,*x*) and *D*(*j*,*x*), where *x* is reaction node index that is not either *i* or *j* are stored and sorted in a vector. *B*(*i*,*j*) is assigned a value between [−1,1] based on the rank of *D*(*i*,*j*) in the sorted vector of distances, computed from a linear formula correlating the shortest distance with a *B*(*i*,*j*) value of 1 and the longest distance with a *B*(*i*,*j*) value of −1. The *B*-matrix was also constructed using parallel computing.

### 4.7. Network Partitioning Using Newman’s Algorithm

The MMA approach uses Newman’s binary partition algorithm to identify modules of reactions that are enriched in metabolites that are statistically significant. The overall algorithm is similar to that described in prior work [30]. Briefly, the complete reaction-centric graph is first checked for connected components, or sub-graphs, as the original Recon 2.1 network is not a completely connected graph network. For each sub-network, a distance matrix D and the modularity B-matrix are constructed as described earlier. Next, a vector *s* of length *N* (number of reactions), comprising of elements −1 or 1 is selected to optimize a modularity score *Q*, which Newman showed is approximated using the leading eigenvector of the B-matrix, where negative entries are denoted as −1 and the positive as +1:

(2) $$Q=\sum_{i}\sum_{j}B_{ij}s_{i}s_{j}$$

After each binary partition, each sub-network is divided into its connected sub-networks if the sub-networks that result from the partition are not completely connected. Partitioning is terminated if the resulting modularity *Q* score for the module is no longer positive.

### 4.8. Random Connected Subnetworks Computation

To obtain SSM density distributions across a network, we performed random sampling of connected sub-networks of a specified size *N* (number of reactions) from the original reaction-centric graph, *G*, and determined the SSM density (number of SSMs divided by the number of reactions) in that subnetwork. The procedure begins by randomly selecting a reaction node from the *G*, and the index of that node is added to a vector denoting the reactions that will comprise the sub-network. Next, a random edge from that node is then selected and the reaction node that the edge connects to in *G* is also added to the sub-network vector. Subsequently, a node is selected at random from the sub-network vector and a random edge attached to that node in *G*. If the new node connecting to the random edge already belongs to the subnetwork, then that edge is removed from *G.* Otherwise, if the new node does not belong to the sub-network, it is added to the sub-network vector. The process is iteratively repeated by picking another node in the sub-network at random in order to grow the connected sub-network until length of the sub-network vector is equal to *N.*

### 4.9. Pathway Enrichment Analysis (PEA)

For each module satisfying the baseline modular criteria, we conducted PEA based on the metabolites in the module to determine if pre-defined KEGG pathways can predict the metabolic interactions discovered by MMA. For a given module, the KEGG ID of each unique SSM in the module is obtained because, in some modules, multiple SSMs may be the same metabolites from different physical compartments. Using the downloaded KEGG metabolite entries downloaded from the server, a list of KEGG-defined metabolic pathways that the metabolite is classified under is obtained. Subsequently, for each unique metabolic pathway represented in the module, we computed the number of modular SSMs that belong to that metabolic pathway. If the number of SSMs belonging to a KEGG pathway is the same as the number of SSMs contained in an MMA-derived module, then it is likely that the metabolic interaction captured by MMA was intuitive based on well-known biochemical pathways. On the other hand, if the maximum number of modular SSMs belonging to the same KEGG pathway is less than the number of modular SSMs, then the interactions captured by the module could not have been determined by just examining classic biochemistry pathways.

### 4.10. Conserved Modules

A module satisfying the baseline modular criteria in the partition of liver X was determined to be conserved in liver Y if a module existed in the partition of Y that contained all the reactions in liver X and also had an SSM density greater than 0.5. If multiple modules in Liver Y exist, the module with the shortest distance to a terminal module in the hierarchical tree was chosen, which will also have the highest SSM density. Indeed, there will always be a module in Liver Y that contains all the reactions comprising a specified module in X since modules close in hierarchical distance to the parent module contain thousands of reactions. However, these modules will have a low SSM density, and, therefore, it is meaningless to claim that the module with a high SSM density in X is conserved in Y based on a module that is close to the parent module. Therefore, we add the constraint that the module in Y must also have a high SSM density. In the first case, this procedure described above was applied to determine all modules in liver X that are conserved in each of the other eight livers. A total of 72 comparisons were made. For the second case, the procedure was applied to determine modules in Liver 1 that were conserved in an un-weighted Recon 2.1 network partition using the metabolomics data for Liver 1.

## Figures and Tables

**Table 1:** Donor characteristics of each discarded liver used for subnormothermic machine perfusion (SNMP). The Warm Ischemia Time (WIT), percent macrovesciular steatosis, age and gender of the donor are listed.

Liver	Classification	WIT	% Steatosis	Age	Gender
1	Control	19	0	54	M
2	Control	19	0	42	F
3	Control	23	0	46	M
4	WI	36	0	35	M
5	WI	44	0	66	F
6	WI	54	0	50	M
7	Steatotic	16	>33%	44	M
8	Steatotic	24	>33%	69	F
9	Steatotic	27	<33%	68	F

**Table 2:** For each liver, the details of the MMA partition are provided, including the number of SSMs, the number of modules with at least one SSM, the number of modules with an SSM density greater than 0.5 and the computational time by running Matlab (version 2015a) on one cluster node (c3.8 × large) with 16 workers on Amazon EC2.

Liver	Num. SSM	Num. MMA Modules	Num. Modules Sig. Met. Density >0.5	MMA Run Time (Hours)
1	73	577	88	2.36
2	75	762	143	2.96
3	92	716	147	2.40
4	65	673	117	2.60
5	87	1061	191	2.33
6	77	544	102	2.76
7	65	748	143	2.51
8	94	894	170	2.53
9	79	790	141	2.67

**Table 3:** For each liver, the number of modules satisfying the baseline criteria and the total number of ‘counter-intuitive modules’ are listed, the latter being defined as a module where no KEGG pathway exists with as many SSMs as contained in the module, suggesting that pre-defined textbook pathways insufficient capture the metabolic interaction.

Liver	Total Modules	Counter-Intuitive Modules	% Total
1	88	20	22.73
2	143	52	36.36
3	147	67	45.58
4	117	41	35.04
5	191	57	29.84
6	102	30	29.41
7	170	91	53.53
8	143	90	62.94
9	141	68	48.23
		Average	40.41
		Stdev	13.03

**Table 4:** For each liver, the number of modules that satisfy the baseline criteria based on edge-weighted network partitioning is listed along with the number of modules that have a corresponding module in the un-weighted partition with the same reaction nodes.

Liver	Total Modules	Number of Corresponding Un-Weighted Modules	% Total
1(25)	88	13	14.77
2(35)	143	9	6.29
3(28)	147	7	4.76
4(34)	117	2	1.71
5(27)	191	9	4.71
6(8)	102	4	3.92
7(30)	170	3	1.76
8(23)	143	5	3.50
9(31)	141	8	5.67
		Average	5.23
		Stdev	3.91

**Table 5:** For each liver, the number of modules that satisfy the baseline criteria based on edge-weighted network partitioning is listed along with the number of modules that contain at each of the four specified cofactors (ATP, NADH, NADPH, FADH).

Liver	Total Modules	NADPH	ATP	NADH	FADH_2_
1	88	8	15	10	3
2	143	20	30	32	0
3	147	21	47	18	2
4	117	16	34	6	2
5	191	21	57	8	1
6	102	6	31	23	1
7	170	23	74	45	7
8	143	17	61	32	1
9	141	21	50	19	1
	Average	17.00	44.33	21.44	2.00
	Stdev	6.08	18.40	12.98	2.06
Unweighted	192	0	3	6	0

## Supplementary Materials

The following are available online at www.mdpi.com/2218-1989/7/4/58/s1, Figure S1: (A) Module featuring alpha-ketoglutarate and 3-hydroxybutyrate, demonstrating that these two metabolites are closely linked based on the shared production/consumption of NADH. (B) The two metabolites would otherwise have been described as being 8 steps away if one were to use the PEA-identified ‘butanoate metabolism’ from KEGG. (C) The corresponding metabolite levels for the module. Figure S2: An example highlighting the necessity of weighting edges based on Reaction Scores to ensure the partition algorithm favors the clustering of reactions that are enriched in Statistically Significant Metabolites, Table S1: Supplementary_Data_Metabolomics_Rawdata, Table S2: Supplementary_Data_Recon_Model, Table S3: Supplementary_Data_Module_Compositions.
