# Supplementary material for: Metabolomic Modularity Analysis (MMA) to Quantify Human Liver Perfusion Dynamics
Source: Metabolites. 2017 Nov 13;7(4):58. doi: 10.3390/metabo7040058 (PMC5746738; doi:10.3390/metabo7040058)
Supplement: Supplementary file 1 [file metabolites-07-00058-s001.zip › metabolites-222986-supplementary/Sridharan_Supplementary_Data_Information_102417.docx]

**Metabolomic Modularity Analysis (MMA) to Quantify Human Liver Perfusion Dynamics**

Gautham Vivek Sridharan^1^, Bote Bruinsma^1^, Shyam Sundhar Bale^1^, Anandh Swaminathan^2^ Nima Saeidi^1^, Martin L. Yarmush^1^, Korkut Uygun^1,*^.

**Supplementary Figures**

**
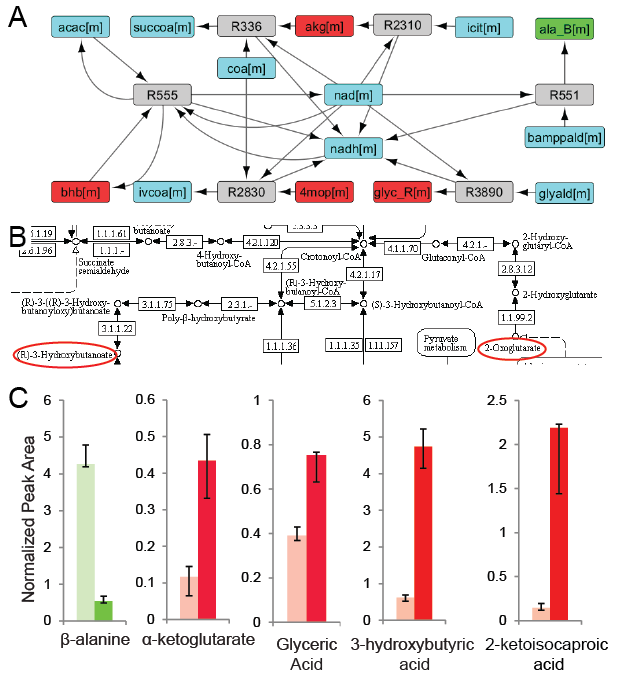
**

Supplementary Figure 1: (A) Module featuring alpha-ketoglutarate and 3-hydroxybutyrate, demonstrating that these two metabolites are closely linked based on the shared production/consumption of NADH, (B) The two metabolites would otherwise have been described as being 8 steps away if one were to use the PEA-identified ‘butanoate metabolism’ from KEGG. (C) The corresponding metabolite levels for the module.


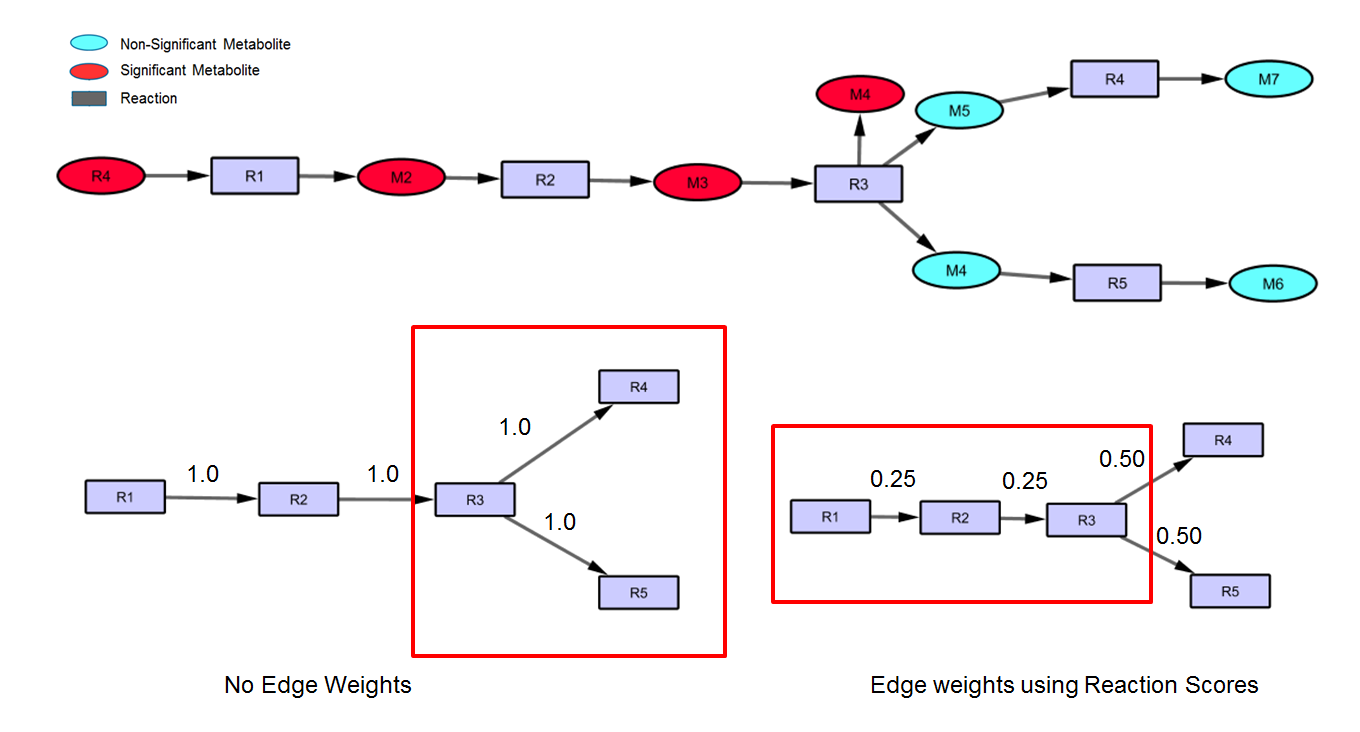


Supplementary Figure 2: An example highlighting the necessity of weighting edges based on Reaction Scores to ensure the partition algorithm favors the clustering of reactions that are enriched in Statistically Significant Metabolites.

**Supplementary Data**

The Supplementary data is presented as spreadsheets as described below:

**Supplementary_Data_Metabolomics_Rawdata.xlsx**

This file has the raw metabolomics data for all livers. The sheet contains the following columns:

1. Met_Name: The common metabolite name
2. KEGG_ID: The KEGG identification number for the metabolite
3. HMDB_ID: The Human Metabolome Database identification number for the metabolite
4. Recon21_Metid: The metabolite ID number in the Recon 2.1 metabolic network. If a metabolite has more than one entry, they are from different intracellular compartments.
5. Recon21_MetNames: The metabolite name listed in the Recon X metabolic network
6. Bipartite_id: The node ID of the metabolite in the bipartite metabolic network
7. Metabolomics_Raw_Data: Raw normalized metabolite peak areas for each liver where the Liver ID is separated by || pipes, each time point is separated by a semicolon ‘;’ and each replicate is separated by a comma ‘,’.

**Supplementary_Data_Recon_Model.xlsx**

The Recon 2.1 Model used for the analysis with the following sheets: The stoichiometric matrix (S_matrix) of the metabolic network (as a sparse matrix), the metabolite and reaction names and IDs, the metabolites that were considered for interactions between nodes (inorganic ions such as H^+^ were not included), the SSMs, the bipartite reaction graph network model, and the reversibility of the reactions. We also provide the sparse matrix of the bipartite-graph, where every interaction is denoted as a ‘1’.

**Supplementary_Data_Module_Compositions.xlsx**

This file contains the composition of each module in the hierarchy for every liver, whose characteristics are described in Table 1 of the main text. For sheets Liver_X_Composition, where X is the ID of the liver, the first column is the module ID, the second column are the IDs of all the reactions, as defined by Recon 2.1, and the last column are the IDs of the SSMs that belong to the module. For sheets Liver_X_hierarchy, where X is the ID of the liver, the first column is the source module ID and the second column is the target module ID in the hierarchical partition for that particular liver. The third column denotes whether or not that source-target relationship is simply a division into the module’s connected components (‘Components’), or whether the relationship is due to a Newman-based binary partition.
